# Biologically-Based Mathematical Modeling of Tumor Vasculature and Angiogenesis via Time-Resolved Imaging Data

**DOI:** 10.3390/cancers13123008

**Published:** 2021-06-16

**Authors:** David A. Hormuth, Caleb M. Phillips, Chengyue Wu, Ernesto A. B. F. Lima, Guillermo Lorenzo, Prashant K. Jha, Angela M. Jarrett, J. Tinsley Oden, Thomas E. Yankeelov

**Affiliations:** 1 Oden Institute for Computational Engineering and Sciences, The University of Texas at Austin, Austin, TX 78712, USA; calebphillips@utexas.edu (C.M.P.); cw35926@utexas.edu (C.W.); ernesto.lima@utexas.edu (E.A.B.F.L.); Guillermo.lorenzo@utexas.edu (G.L.); prashant.jha@austin.utexas.edu (P.K.J.); oden@oden.utexas.edu (J.T.O.); Thomas.yankeelov@utexas.edu (T.E.Y.); 2 Livestrong Cancer Institutes, Dell Medical School, The University of Texas at Austin, Austin, TX 78712, USA; 3 Texas Advanced Computing Center, The University of Texas at Austin, Austin, TX 78758, USA; 4 Department of Civil Engineering and Architecture, University of Pavia, Via Ferrata 3, 27100 Pavia, Italy; 5 Department of Biomedical Engineering, The University of Texas at Austin, Austin, TX 78712, USA; ajarrett@utexas.edu; 6 Department of Aerospace Engineering and Engineering Mechanics, The University of Texas at Austin, Austin, TX 78712, USA; 7 Department of Mathematics, The University of Texas at Austin, Austin, TX 78712, USA; 8 Department of Computer Science, The University of Texas at Austin, Austin, TX 78712, USA; 9 Department of Diagnostic Medicine, The University of Texas at Austin, Austin, TX 78712, USA; 10 Department of Oncology, The University of Texas at Austin, Austin, TX 78712, USA; 11 Department of Imaging Physics, The University of Texas MD Anderson Cancer Center, Houston, TX 77030, USA

**Keywords:** computational oncology, magnetic resonance imaging, perfusion, partial differential equations, confocal microscopy, systems biology, treatment response, vascular growth, computational fluid dynamics

## Abstract

**Simple Summary:**

The recruitment of new vasculature via angiogenesis is a critical component of tumor development, which fundamentally influences tumor growth and response to treatment. The characterization of tumor-induced angiogenesis via mathematical models could enable approaches to forecast tumor response and improve patient care. In this review, we discuss how time-resolved imaging data integrated with mathematical modeling can be used to systematically investigate angiogenesis from the cell to tissue scale and ultimately forecast response to therapy.

**Abstract:**

Tumor-associated vasculature is responsible for the delivery of nutrients, removal of waste, and allowing growth beyond 2–3 mm^3^. Additionally, the vascular network, which is changing in both space and time, fundamentally influences tumor response to both systemic and radiation therapy. Thus, a robust understanding of vascular dynamics is necessary to accurately predict tumor growth, as well as establish optimal treatment protocols to achieve optimal tumor control. Such a goal requires the intimate integration of both theory and experiment. Quantitative and time-resolved imaging methods have emerged as technologies able to visualize and characterize tumor vascular properties before and during therapy at the tissue and cell scale. Parallel to, but separate from those developments, mathematical modeling techniques have been developed to enable in silico investigations into theoretical tumor and vascular dynamics. In particular, recent efforts have sought to integrate both theory and experiment to enable data-driven mathematical modeling. Such mathematical models are calibrated by data obtained from individual tumor-vascular systems to predict future vascular growth, delivery of systemic agents, and response to radiotherapy. In this review, we discuss experimental techniques for visualizing and quantifying vascular dynamics including magnetic resonance imaging, microfluidic devices, and confocal microscopy. We then focus on the integration of these experimental measures with biologically based mathematical models to generate testable predictions.

## 1. Introduction

In the early stages of tumor growth, a small population of tumor cells is supported by existing tissue vasculature and the diffusion of nutrients through the extravascular space. As this small population of tumor cells continues to grow, it may eventually reach a size where the diffusion of nutrients from existing vasculature is insufficient to support continued growth. Through the process of angiogenesis, new blood vessels are recruited from nearby vasculature to provide the crucial infrastructure needed to sustain further expansion of the tumor [1]. These three key points inform the foundation of many mathematical models of angiogenesis and arose from the seminal work on tumor angiogenesis by Folkman [1,2] and others [3,4] over the past half a century. Additional studies on angiogenic signaling [5,6] and vasculature properties [4,7] have also greatly influenced the development of a mathematical theory on angiogenesis. One notable observation was that compared to healthy appearing vasculature, tumor-associated vasculature has substantial structural and functional abnormalities characterized by non-hierarchical vessel networks, heterogeneous blood flow, and heterogeneous permeability [4,7]. These irregularities significantly influence the delivery of nutrients and removal of waste, while also having substantial implications on systemic and radiation therapy [8].

Tumor vasculature and the process of angiogenesis have a critical and complex role in the response of solid tumors to systemic therapies. First, the successful delivery of systemic agents is contingent on functional vasculature providing a homogenous delivery of therapeutics. Unfortunately, the abnormal structure and function of vessels associated with tumor-induced angiogenesis yields a heterogenous distribution of therapeutics (which can include chemotherapies as well as targeted and hormone therapies) throughout the tumor, contributing to varied efficacy within patient populations. For example, in breast cancer, clinical trials for neoadjuvant systemic therapies have resulted in only 6–26% of patients achieving a pathological complete response by the completion of treatment, which, in part, may be due to the abnormal structure and development of the vessels [9]. Second, the vasculature itself can also be affected by targeted and non-specific systemic therapies that may hinder angiogenesis, eliminate vasculature, or normalize existing neovasculature [10]. One perspective on cytotoxic agents inflicting damage to tumor-associated vasculature is that it is a positive outcome, which can prevent necessary nutrients from reaching the tumor and to induce necrosis. An opposing view, however, posits that efforts should be made to protect (and even normalize) the vessels to enhance the delivery of the therapeutics [10,11]. Therefore, tumor-associated vasculature plays an important and evolving role in the effectiveness of systemic cancer treatment.

Radiotherapy is another primary treatment option for the majority of solid tumors and is capable of targeting unresectable or highly invasive disease. The efficacy of radiotherapy, however, is highly dependent on the structure and function of the tumor-associated vasculature. For over half a century, it has been well-known that tissue oxygenation influences the sensitivity of tumor cells to radiotherapy [12]. Within tumors, tissue oxygenation is highly heterogeneous due to the structural and functional abnormalities of tumor vasculature, which can result in both acute and chronic hypoxic regions resistant to radiotherapy [13]. Large hypoxic regions also occur from vascular injury or vascular occlusion—often downstream of increased mechanical pressures from increased tumor cellularity [14]. It is generally presumed that tumor cells nearest to functioning vasculature and furthest from hypoxic regions are often the most responsive to radiotherapy. Thus, after radiotherapy, the remaining tumor is thought to be largely composed of poorly perfused and hypoxic tumor cells. However, radiotherapy itself also influences tumor-associated vasculature by promoting angiogenesis, the revascularization of the remaining tumor, and the reoxygenation of tumor tissue—thus improving the sensitivity of previously hypoxic cells to future doses of radiotherapy [15,16]. To effectively control tumors via radiotherapy and identify optimal radiotherapy regimens, knowledge of the dynamic relationship that exists between tumor-associated vasculature and radiotherapy is required.

It is clear that tumor vasculature and angiogenesis significantly influence tumor growth and response to systemic and radiation therapies. To improve patient outcomes, therapeutic regimens need to be optimized while considering the structural and functional characteristics of an individual’s tumor-associated vasculature. Achieving this goal requires a biophysical mathematical theory that accurately characterizes the relevant quantities of interest in the dynamic relationship between the tumor, vasculature, and therapy. Given such a theory, one could identify, through systematic, in silico evaluations, therapeutic regimens that are personalized to optimize treatment outcomes for each individual patient [17]. While the literature is filled with numerous theoretical studies characterizing tumor-associated vasculature from the cell to tissue scale, there is a lack of research that explicitly links theory with quantitative experimental studies [18,19]. 

Quantitative and time-resolved imaging approaches, such as confocal imaging, photoacoustic imaging, and magnetic resonance imaging (MRI), could provide the necessary data to initialize, calibrate, and validate models of angiogenesis. Specifically, time-resolved imaging techniques of the vasculature have matured to the point where they can define or estimate subject-specific structural (e.g., vessel order and location) and functional (e.g., vessel permeability, blood flow) model parameters that would enable in silico investigations of tumor and vasculature dynamics [17]. Non-invasive imaging techniques preserve the system under observation, allowing the state of the system to be assessed and quantified before, during, and after treatment, thereby capturing the evolution of both tumor and vasculature. This spatially and time-resolved data is a fundamental component of rigorous model development and validation that is required to translate modeling approaches (and predictions) to the clinic [20,21].

The mathematical modeling of tumor angiogenesis at the cell scale has developed into a rich literature over the last few decades [22,23,24,25,26,27]. These models aim to give a rigorous mathematical description of tumor angiogenesis to enable the systematic investigation of the underlying biology that dictates vascular sprouting, perfusion, and response to therapy. By employing such models, it is possible to simulate and test scenarios in silico that are not easily tested experimentally. For example, comparing the limitless number of therapeutic regimens that can be constructed with varying dosing schedules and concentrations is experimentally intractable, but with a mathematical model these can be simulated and analyzed to select the optimal regimen [28]. Recently, there has also been great interest in the modeling of tumor angiogenesis at the tissue scale [29,30,31]. These efforts have often been motivated by the emerging availability of crucial vasculature properties in patient or animal data that previously could only be assessed through highly invasive means—such as angiogenesis and regression rates [32] (i.e., a time scale of formation and regression of tumor-induced vasculature), interstitial pressure, and blood pressure along vessels [33]. However, these models must be informed and validated by time-resolved, experimental data to initialize and calibrate key model parameters, or by modeling biologically based hypotheses and testing model output with experimental observables.

In this review, we identify promising approaches that integrate mathematical theory with experimental data from the in vitro cell scale to in vivo tissue scale, discuss opportunities for bridging cell and tissue scale models, and present future opportunities for applying these models to optimize therapeutic regimens and therefore improve patient care. To prepare this review, we first identified literature that integrated mathematical theory with experimental imaging data. We then identified reviews or landmark articles that provided the foundation for both the mathematical theory and experimental techniques.

## 2. Overview of Experimental Techniques across Scales

**Figure 1:**
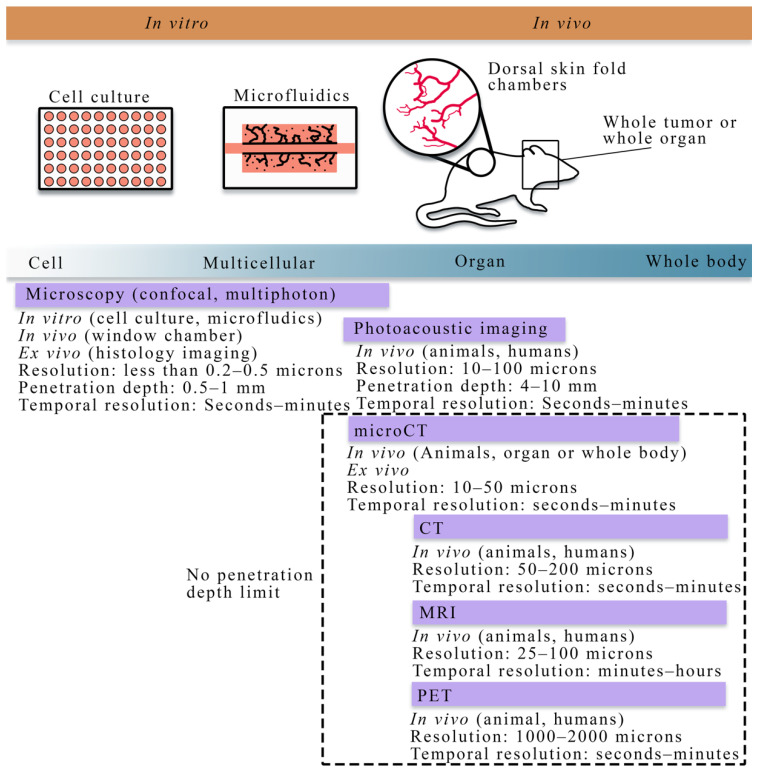
Overview of cell to tissue scale imaging. Experimental platforms from the cell to tissue scales consist of cell culture (to investigate cell dynamics), microfluidics (a perfused cell culture platform to observe angiogenesis), skin fold window chambers (an in vivo platform for optical imaging), and small animal or human whole organ and body imaging (for in vivo studies). Imaging techniques (purple bars) vary across spatial and temporal scales. In vitro imaging consists primarily of the microscopies (e.g., confocal, multiphoton). In vivo imaging is achievable with all the imaging techniques shown above; however, there are limitations in the penetration depth for microscopy and photoacoustic imaging. Magnetic resonance imaging (MRI), positron emission tomography (PET), and computed tomography (CT) are primarily in vivo techniques capable of whole animal or human imaging. Whole animal or body imaging is feasible with microCT, though it is typically used for whole organ or ex vivo imaging.

In this section, we discuss experimental techniques from the cell to tissue scales. Figure 1 summarizes the cell to tissue scale approaches for imaging experiments, while Table 1 lists the imaging techniques and the literature that integrates those techniques with mathematical theory at the cell and tissue scale. We note that the literature listed in Table 1 is presented in detail in Section 3.2 and Section 4.2 for the cell and tissue scale, respectively.

### 2.1. Quantitative Techniques for Observing Tumor Vasculature and Angiogenesis at the Cellular Scale

At the cellular scale, microscopy is the dominant imaging technique for providing quantitative measurements of tumor vasculature with a spatial resolution on the order of microns. Confocal microscopy, combined with immunofluorescence staining, has been used to acquire high resolution, temporally resolved images of vascular structure in angiogenic and vasculogenic assays [34,35,36]. This technology allows for analyzing in vitro spatial distributions of fluorescently labeled cell lines and can be coupled with fluorescing microspheres to investigate vascular integrity, enabling the integration of quantitative fluorescence measurements with mathematical modeling. Furthermore, confocal microscopy has also been utilized in vivo to investigate functional microcirculation [37] in tumor-associated vasculature, the effects of radiotherapy [38] on neovasculature, and the oxygen distribution [39,40] in dorsal skin fold chambers [41]. While intravital microscopy provides a high-resolution longitudinal analysis in vivo, the chamber may alter the tumor-vasculature dynamics and it is fundamentally limited in the length of study (generally 2–3 weeks), and the number of imaging time points. Multiphoton microscopy [42], in comparison to confocal microscopy, has improved depth penetration and confines excitation to the focal plane of the lens, thereby decreasing the photodamage to the tissue. For many biological applications, tissue depths of ~500 microns can be imaged over time [42].

In addition to microscopy, photoacoustic imaging offers high spatial resolution (10–100 microns) while also being able to reach tissue penetration depths of around 4–10 cm [43,44]. Photoacoustic imaging probes the tissue of interest with pulses of light, creating changes in the pressure when the light is absorbed. These changes in pressure generate ultrasound waves that are detected at the tissue surface. The spatial and temporal resolution, imaging depth, and image contrast can be selected by utilizing different light sources, ultrasound wave detectors, and scanning methods to cater to the specific application under study, making photoacoustic imaging a promising emerging imaging modality at the cellular scale [43].

### 2.2. Quantitative Techniques for Observing Tumor Vasculature and Angiogenesis at the Tissue Scale

All the techniques presented in this section are suitable for small animal and human imaging. Ex vivo and in vivo imaging play a central role in understanding the morphology and function of tumor vasculature and angiogenesis. In particular, ex vivo imaging techniques, including histology imaging [45] and micro-CT [46,47,48], can quantify tumor microvasculature and angiogenesis on excised tissue specimens and serve as the gold standard measurement. Less invasive observations of tumor-associated vasculature can now be achieved thanks to the development of in vivo imaging techniques [49], such as x-ray [50] and computed tomography (CT) [51,52,53], positron emission tomography (PET) [54,55], MRI [52,56,57,58,59,60,61,62], and optical imaging [63,64]. There are two main classes of imaging techniques applied to studying tumor-associated vasculature: (1) angiography, which is a technique used to visualize the vasculature structure, and (2) functional techniques used to quantify the properties of the tissue and vasculature. In conventional planar x-ray angiography [50], the patient is catheterized so that an iodinated contrast agent can be administered intravenously and then observed with fluoroscopy, thereby enabling the observation of the vascular architecture. CT angiography is an extension of x-ray angiography that enables the visualization of vessel structures in 3D. CT angiography has been commonly used to identify the location and anatomy of tumor-associated vessels (especially for pancreatic tumors), which plays a valuable role on diagnosis and the management of chemotherapy and surgery [65,66,67]. Magnetic resonance angiography (MRA) is an alternative approach that does not use ionizing radiation and can be used to visualize blood vessels, especially large arteries and veins [56]. MRA techniques aim at enhancing the contrast between blood vessels and the background tissue based on either the effects of blood flow on MR signal or the injection of exogenous contrast agents, thereby allowing for the quantification of several morphological characteristics of the vasculature, such as vessel tortuosity, density, diameter, and branching patterns as well as feeding and draining vessels [68,69].

While conventional angiography focuses on vascular morphology, functional imaging techniques enable the extraction of information regarding hemodynamics and pharmacokinetics. CT-based techniques have been developed to provide physio-pathological information of the vasculature beyond the anatomy [51,70]. Functional or dynamic contrast-enhanced (DCE-) CT can measure tumor vascular features including blood flow, blood volume, mean transit time, and permeability-surface area product. Functional CT could potentially be used to monitor the change of tumor perfusion in anti-angiogenic therapy [70]. CT has also been combined with PET techniques for the staging and monitoring of multiple types of tumors via the evaluation of blood flow, such as melanoma, medullary thyroid cancer, hepatocellular carcinoma, and prostate carcinoma [54,55]. Similar to the functional CT approach, DCE-MRI can also return estimates describing plasma volume fraction, extracellular extravascular volume fraction, and vessel permeability and perfusion. DCE-MRI techniques with high temporal resolution (7 s per frame or even faster) [71,72] further enable the extraction of information regarding hemodynamics and pharmacokinetics. Recent studies on the hybrid acquisition of MRA and DCE-MRI allow for the extraction of both morphological and functional features of tumor-associated vasculature, which have been shown to increase the diagnostic accuracy of suspicious tumors [73]. Although in vivo imaging provides an observation of vasculature non-invasively, these technologies are significantly limited by the available spatial and temporal resolution and their signal-to-noise ratio. Thus, the common, clinically available angiography techniques cannot currently capture details of the microvasculature. To overcome this limitation, the development of window chamber models could be a promising approach [74,75], as this technology enables the combination of in vivo microscopy with MR imaging, thereby potentially enabling the validation of macroscopical measurements of microvasculature via MRI.

**Table 1:** Imaging techniques for visualizing vasculature and angiogenesis at cell scale and tissue scale.

Modality	Scale	Measurement	Uses in Literature
Microscopy (confocal, multiphoton, optical projection tomography, histology imaging)	Cell to tissue	Vascular structure, individual cell types, vessel porosity, flow	[18,76,77,78,79,80]
Photoacoustic imaging	Cell to tissue	Vascular structure, blood oxygenation	[81,82]
Angiography (X-ray, CT, MRI)	Tissue	Vascular structure	[47,48,83,84]
Dynamic contrast-enhanced MRI or CT	Tissue	Perfusion, permeability, blood volume fraction	[28,32,33,85,86]
PET	Tissue	Perfusion, permeability, blood volume fraction	[83,84]
microCT	Tissue	Vascular structure	[47,48,83,84]

## 3. Approaches for Modeling Tumor Vasculature at the Cell Scale

In this section, we identify a few landmark mathematical, cell scale models of tumor vasculature and angiogenesis. We then describe the common quantitative methods for observing angiogenesis over time and conclude by discussing some efforts, both established and ongoing, to integrate mathematical models with experimental methods.

### 3.1. Mathematical Modeling of Tumor Vasculature and Angiogenesis at the Cell Scale

**Figure 2:**
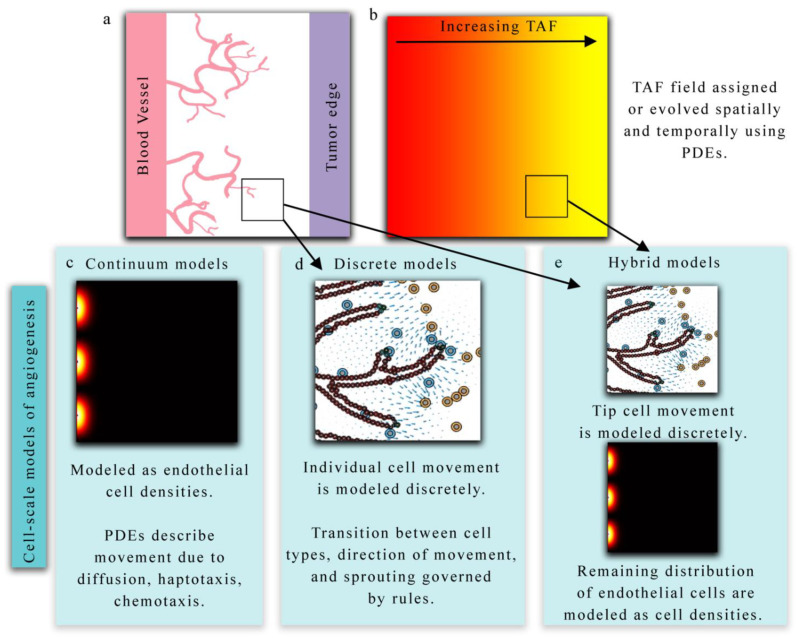
Overview of cell-scale models of angiogenesis. (Panels **a** and **b**) present a hypothetical biological scenario in which new vasculature is recruited via angiogenesis in response to tumor angiogenic factors (TAF) released by tumor cells. Continuum models (panel **c**) describe this phenomenon in terms of endothelial cell densities and the concentration of TAF. Partial differential equations (PDEs) provide a continuous representation of endothelial densities and often describe the spatial and temporal evolution via diffusion, haptotaxis, and chemotaxis terms. Alternatively, discrete models (panel **d**) can be used to explicitly describe the movement and behavior of each individual endothelial cell. Hybrid models (panel **e**) generally combine both discrete and continuum approaches to model TEC movement and endothelial cell densities, respectively, in response to TAF.

Mathematical models of tumor angiogenesis vary in the extent of biological detail they characterize and can be summarized as discrete (treating endothelial cells and vasculature as individual objects), continuous (treating endothelial cells or vasculature as concentrations), or hybrid (combining methodology from both discrete and continuum theory) models. Discrete models may track all endothelial cells as individual agents, or simply tip endothelial cells or TECs (the cells responsible for directed migration in response to chemical stimuli). In discrete models, the vasculature changes through time based on sets of rules dictating cell behavior (e.g., whether a cell will divide or migrate). Continuum models are based on ordinary or partial differential equations (PDEs) that govern the behavior of the endothelial cells through time. Hybrid models couple these two theories by, for example, discretely characterizing the TECs and continuously modeling the overall vessel morphology through a PDE. We note that while hybrid models could refer to models that have a discrete and continuous component within the modeling framework (which would dictate a hybrid modeling approach), here, we define hybrid as utilizing both discrete and continuous methodologies specifically applied to model the vasculature. The reader is invited to refer to Figure 2 throughout this section as it shows examples of these three modeling approaches. All three modeling approaches are typically used to study the migration and development of tumor-associated vasculature in response to external stimuli (e.g., chemical, mechanical) in conjunction with a model of tumor growth. A simulation of the process of angiogenesis typically begins with the stimulation of endothelial cells by tumor angiogenic factors (TAF, a continuous field of pro-angiogenic proteins secreted by tumor cells) that are either explicitly coupled to a model of tumor cell growth or assumed to have a fixed initial distribution. Directed movement of endothelial cells is then influenced by chemical gradients (i.e., TAF), gradients in fibronectin or insoluble extra-cellular matrix (ECM) (i.e., haptotaxis), and mechanical cues (i.e., mechanotaxis) [87,88].

#### 3.1.1. Continuum Models

**Figure 3:**
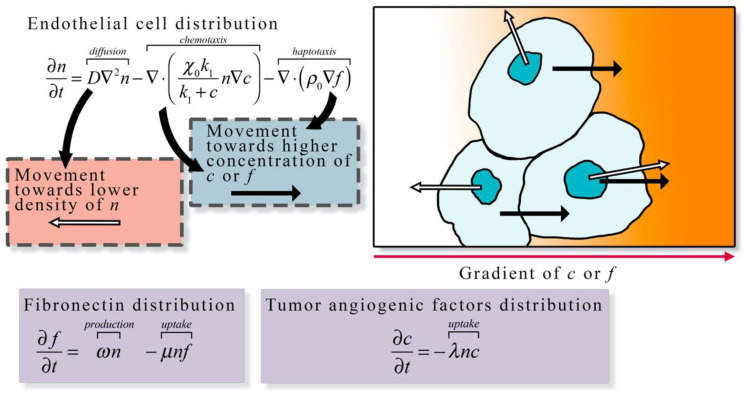
Continuum description of angiogenesis. The continuum description of angiogenesis developed by Anderson et al. [23] describes the spatial and temporal change in endothelial cell density (*n*) as the function of diffusion, chemotaxis along tumor angiogenic factor (*c*) gradients, and haptotaxis along fibronectin (*f*) gradients. Endothelial cell diffusion is characterized by a diffusion coefficient *D*, chemotaxis is characterized by chemotaxis coefficients $\chi_{0}$ and *k*_1_, and haptotaxis is characterized by the haptotaxis coefficient $\rho_{0}$. In the presence of other cells endothelial cell movement via diffusion is directed away from high densities of *n* (white arrows in the illustration), otherwise the movement via diffusion is random. Both chemotaxis and haptotaxis result in endothelial cell movement towards higher concentration of *c* or *f* (black arrows in the illustration), respectively. The change in fibronectin distribution over time is the function of the production at rate *ω* by endothelial cells and the uptake at rate *μ* by endothelial cells. The change in tumor angiogenic factor distribution is described by the uptake at rate *λ* by endothelial cells. The general formulation of the left-hand side of the equation expressing the rate of change of a quantity of interest, and the right-hand side describing all the ways it can change is frequently the over-arching guide for constructing such models.

Continuum models (panel c in Figure 2) describe the spatial and temporal development of endothelial cells over time in terms of densities or volume fractions [89,90,91]. These models are capable of capturing macroscopic features related to vasculature, TAF, and ECM but do not track individual cells or vessel segments. In continuum models, the spatial and temporal progression of these model components are described with a set of coupled PDEs. Anderson et al. developed a continuum model of tumor angiogenesis by considering the rate of change of endothelial cell density determined by the sum of the effects of Brownian motion (diffusion), chemical stimuli (chemotaxis), and mechanical forces (haptotaxis) [23]. The chemical stimuli considered was TAF, which caused a migration in endothelial cell density toward the TAF source. In the presence of angiogenic factors, the distribution of endothelial cells migrated across the domain. As Anderson et al.’s model system forms the foundation for numerous other models of angiogenesis, Figure 3 illustrates the proposed model. The same model can be conceptualized as a discrete model by considering the bulk changes in endothelial cell density as discrete events based on probabilities. The authors used a finite difference approximation of the continuous equation for endothelial cell density to determine the probability that endothelial cells move in a particular direction due to diffusion, chemotaxis, and haptotaxis. This work highlighted the potential of both discrete and continuum models to explore the same phenomena.

#### 3.1.2. Discrete Models

Discrete models (panel d in Figure 2), however, specifically track individual endothelial cells rather than densities. Discrete models can be divided into two main categories: lattice-based and lattice-free. Lattice-based methods allow cells to migrate or divide according to a gridded system (i.e., the lattice), where each cell may occupy one or many lattice sites, while lattice-free methods (or agent-based methods) allow cells to freely migrate and divide in any direction. Lattice-based methods where a cell occupies one lattice site are called cellular automaton models [76,92,93], while models where cells occupy many lattice sites are called Cellular Potts models or CPMs [94,95]. Cellular automaton models use a structured lattice where each cell occupies one lattice site and cells are updated through time as they move (from one lattice site to another), proliferate (cell divides and places a daughter cell in a neighboring site), or die (removal of a cell within a lattice site). A landmark cellular automaton model by Anderson et al. was extended in McDougall et al. [96] to describe vessel formation, loop formation (anastomosis), and blood flow through the vasculature. They utilized a Poiseuille-like expression for blood flow that is dependent on vessel radius, where the radius adapts based on wall shear stress, intravascular pressure, and metabolic stimuli. These additions allow for the simulation of blood flow through dynamically remodeling vessels that subsequently affects the delivery of both nutrients and therapeutics. Owen et al. [92] developed a multi-scale cellular automaton model to describe the evolution of vasculature through angiogenesis and vascular pruning due to low wall shear stress. A subcellular scale model describing cell cycle, apoptosis, and vascular endothelial growth factor (VEGF) secretion was coupled to a cellular scale model describing the movement and interaction between normal, tumor, and endothelial cells. Both the subcellular and cellular scale models were coupled to continuum models applied to diffusible species (e.g., VEGF and oxygen). In their approach, the level of tissue oxygenation drives normal cells to produce VEGF and stimulate endothelial sprouting. The authors applied their model to study angiogenesis and vascular remodeling under different initial vasculature networks, and observed that if the vasculature network was sparse the tumor would remain localized until new vessels are formed [92].

An alternative lattice-based approach is the CPM. In CPMs, cells may occupy several lattice sites, and each cell is identified by a unique lattice index. Therefore, lattice sites with different lattice indices are occupied by different cells. Neighboring cells form connections between each other and share an adhesive bond energy. CPMs are designed to minimize the energy of the system, where the effective energy is the sum of all the bond energies between cells and the differences between the volume of each cell and the target volume of a cell (this energy results from a cell’s resistance to volumetric changes). The effective or total energy is captured by the Hamiltonian which is an operator that is the sum of energies describing the modeled biological processes (e.g., chemotactic energy, haptotactic energy, cell division energy). A typical CPM algorithm is as follows: (1) a random lattice site *i* is selected, (2) a neighboring lattice site *j* is selected and is changed to the same index as site *i*, (3) the Hamiltonian is calculated for this new configuration, and (4) if the energy decreases compared to the original configuration the site retains the new index otherwise it reverts to its original index. By changing the Hamiltonian describing biological systems, CPMs have become a mainstay in modeling tumor angiogenesis and endothelial cell arrangement. In Merks et al. [97], the authors utilized a CPM to model vascular organization with and without contact inhibition between endothelial cells and displayed the ability of the model to recapitulate vessel networks with various morphologies. They included a term modeling chemical signaling based on the concentration of a generic chemoattractant (such as VEGF) around the endothelial cells, causing a shift in the energy to promote angiogenic sprouting. This energy formulation is coupled to a PDE of the chemoattractant describing its secretion by endothelial cells, its diffusion throughout the microenvironment, and its decay over time.

Lattice-free methods [98], or agent-based models, allow cells to migrate and divide in any direction and are not constrained by an underlying lattice. In Plank et al. [99], an off-lattice method is developed by considering TEC migration to be based on the turning rate of a cell (i.e., the rate at which a cell changes its orientation) and the preferred migratory direction along the gradient of TAF. They compared the resulting vasculature simulated from the lattice-free model with the results of several on-lattice models. Notably, the networks generated by the off-lattice model had a higher tendency to form anastomosis loops and had less orthogonal jumps, a common feature in lattice-based models. Phillips et al. [100] developed an agent-based model of tumor-induced angiogenesis, where endothelial cells are activated by TAF, which is modeled as a continuous field through a PDE. The activated cells transition to TECs that migrate up the concentration gradient of TAF and cause neighboring cells to adopt a stalk phenotype, described by rapid proliferation to allow the extension of the angiogenic sprout. These cells interact through mechanical forces that establish lumen stability and allow for an angiogenic network to form. Additionally, the physical interaction between the tumor and the new vasculature network is included and allows the tumor to collapse vasculature segments and reduce nutrient delivery.

#### 3.1.3. Hybrid Models

Hybrid models (panel e in Figure 2) [100,101,102,103,104,105,106,107] combine discrete and continuum methodologies by (generally) describing the TECs as discrete agents that migrate chemotactically in the presence of a TAF gradient and a PDE model describing endothelial cell density. These models seek to take advantage of fast model computations when solving continuous PDE models, but also have a more robust description of specific cell actions. In Lima et al. [107], TECs are modeled discretely and move according to the extracellular matrix conductivity, a chemotaxis constant, and the gradient of TAF, while the endothelial cell volume fraction is updated based on the movement of the TEC. In Vilanova et al. [101], capillaries are modeled using a continuum approach describing the movement, proliferation, and apoptosis of the cells within the capillaries. TECs are identified within the field of capillaries based on the concentration of TAF and lateral inhibition (no TECs are within a distance threshold of the cell to be activated). The model is analyzed by considering scenarios of the growth phase of angiogenesis, chemical inhibition through therapeutics, and the reinitiating of vessel growth after removing chemical inhibition.

#### 3.1.4. Summary

The continuum, discrete, and hybrid modeling approaches above provide complementary information on angiogenesis, and the choice of modeling approach is dependent on the desired goal or quantity of interest from the model itself. The primary advantage of using a continuous representation of tumor vasculature is the low computational cost, and the ability to utilize sophisticated parallel solvers for continuum equations. However, a continuum approach lacks the ability to resolve local key features of the changing vasculature including, for example, the activation of TECs (the cell responsible for directed migration) and the competition for the TEC phenotype among other TECs and neighboring endothelial cells. Discrete models can resolve these local features but become computationally expensive as the number of cells increase. Hybrid models balance both approaches and produce robust and sophisticated vascular fields, but often require complex numerical schemes to solve them. All three modeling approaches have been shown to qualitatively describe the dynamics of tumor angiogenesis; however, many parameters in these models are often assigned values without any experimental validation. This leads to models matching qualitative properties of angiogenesis such as TEC activation, sprout elongation, formation of anastomosis, and establishing blood flow, but have difficulty predicting actual experimental outcomes, since parameters are freely assigned. Recent advances, though, indicate that time-resolved quantitative imaging can provide the data necessary to inform and calibrate model parameters specific to the vasculature network under investigation.

### 3.2. Integrating Theory and Experimental Data at the Cellular Scale

Integrating mathematical models and experimental data has the potential to yield a set of validated models that can then be used to make specific predictions in silico. These model predictions can then be rigorously tested experimentally. However, to date, there has been a paucity of published examples that rigorously calibrate mathematical models to experimental data of tumor angiogenesis at the cellular scale. This is due to complexities in both the computational and experimental efforts, and the difficulties in integrating the two. Computational complexities include the sophisticated numerical schemes that must be used to solve mathematical models of angiogenesis at the cell scale, (which can be very expensive to solve), the necessity of these numerical schemes to be fast enough to calibrate model parameters (which can take thousands of model runs), and ensuring that calibrated model parameters drive the system (since uncalibrated or free model parameters cannot be trusted to generate reliable model predictions). Experimental complexities include the necessity of reproducible, quantitative, high resolution, longitudinal imaging that can isolate processes critical to angiogenic sprouting and tumor vasculature. Microfluidic devices are one promising platform that enable the culturing of tumor and/or endothelial cells in 2D or 3D, while simultaneously incorporating biochemical gradients, fluid flow, and mechanical signaling [108,109]. These devices can play a powerful role in the study of tumor angiogenesis and vasculature by providing a controlled, repeatable experimental platform in vitro that can isolate specific processes that are not easily studied individually in vivo. Many microfluidic devices are widely reproducible and allow for a systematic investigation of vasculogenesis [110,111,112,113], angiogenesis [114,115,116,117], and response to anti-angiogenic therapies [118].

While computational advances in discrete, continuum, and hybrid modeling along with experimental advances in microscopy and microfluidic devices have largely bridged this gap, significant progress in the rigorous integration of mathematical models of angiogenesis and experimental observations have yet to be realized at the cell scale. We now highlight some promising approaches that integrate in vitro and in vivo experiments with mathematical theory.

Perfahl et al. [76] extended a 2D multiscale model of vascular tumor growth, coupling blood flow, tumor-induced angiogenesis, and vascular remodeling in Owen et al. [92] to 3D and initialized the model with vasculature imaged in an in vivo mouse model. To observe angiogenesis in vivo, a murine dorsal skin fold chamber was implanted with a 1 cm diameter glass coverslip and imaged after the mouse was inoculated with red fluorescing tumor cells and green fluorescing microvessels. The resulting vasculature network was imaged using multiphoton microscopy, with z-stacks (i.e., images acquired at different focal distances) acquired at 0.5 µm intervals. The z-stacks were then reconstructed to produce a 3D volume of vasculature, which was used to initialize the vasculature position in the mathematical model. Their angiogenesis model utilized a cellular automaton approach, where tumor cells release TAF that diffuses through the microenvironment and induces angiogenesis. Their approach was used to study how different initial vasculature networks influenced tumor growth dynamics.

Xu et al. [81,82] developed a 3D hybrid model of tumor angiogenesis coupled to TAF, interstitial flow, and blood flow, which was initialized with photoacoustic imaging data. TAF dynamics were modeled with a reaction-diffusion PDE describing the secretion of TAF by hypoxic tumor cells, diffusion through the extracellular space, uptake by endothelial cells, and decay of TAF over time. Photoacoustic images were obtained from [44] in a murine xenograft with an imaged volume of 14 mm × 14 mm × 6 mm (depth) over 26 days. While the primary purpose of this experimental study is to investigate a novel photoacoustic contrast methodology, the authors yield longitudinal images of tumor-associated vasculature at depths approaching 10 mm and a spatial resolution of under 100 microns. Photoacoustic images were scaled between −1 and 1 to segment extravascular space and capillaries, respectively. The map of capillaries and extravascular space was then used as the initial vasculature network in the model by Xu et al. [81]. While the model was not calibrated by time-resolved data, their image processing and modeling framework demonstrated an approach to readily utilize photoacoustic imaging data directly in the model without extensive image processing or manual adjustments.

In Stepanova et al. [77], a multiscale cellular automaton model for angiogenesis was developed and compared to experimental data using the displacement, orientation, and directionality of endothelial cells across multiple concentrations of VEGF. The displacement, orientation, and directionality of endothelial cells was calculated in the model and compared with experimental values from longitudinal confocal microscopy images collected every 15 min for 36 h, under concentrations of 0 ng/mL, 5 ng/mL, and 50 ng/mL of VEGF. These estimates of the displacement, orientation, and directionality of endothelial cells were used to calibrate model parameters. The calibrated model parameters were used to simulate characteristic features of angiogenic sprouting such as branching, chemotactic sensing, the brush border effect, and cell mixing. Additional model validation was done by designing numerical simulations that recapitulate the experiments shown in Jakobsson et al. [78]. Competition between wildtype endothelial and mutant endothelial cells (e.g., heterozygous for VEGF-1 and exposed to a Notch signaling inhibitor) for the TEC position was summarized by the percentage of time each cell line is in the lead cell position (acting as a TEC). In the experimental setup, wildtype and mutant cells are fluorescently labeled red and green, respectively, and time-lapse confocal microscopy of mosaic embryoid body cultures was done over periods of 1 to 4 days. Image segmentation and cell tracking ultimately provided the percentage of time each cell line acts as the TEC. Equivalent measurements from Stepanova et al.’s cellular automaton model were compared directly to the experimentally observed behavior and agreed with Jakobsson et al.’s [78] studies.

Phillips et al. [18,79,100] have proposed integrating confocal microscopy data from an in vitro vascularized tumor platform [117] with an agent-based mathematical model of tumor angiogenesis [100]. In their framework, time-resolved confocal measurements of individual angiogenic sprouts are used to calibrate and validate a multiscale agent-based model. The agent-based model captures the dynamics of endothelial cells. Each agent represents a single endothelial cell that can be in one of the following phenotypes: tip, stalk, or phalanx cell [107]. Tumor cells release TAF, which is modeled by a reaction-diffusion equation and is responsible for guiding the movement and phenotypic transitions of endothelial cells. In their preliminary study [79], they calibrated the endothelial cell cycle duration and TEC velocity and used these parameters to estimate the total sprout length at the end of the imaging experiment. Phillips et al. [79] observed a 12.5% error in sprout length between the model and the image measurement. Future efforts are aimed at improving the spatial agreement between the model and the measurements. Table 2 summarizes the literature reviewed in this section and how the selected models are integrated with imaging data.

## 4. Approaches for Modeling Tumor Vasculature and Angiogenesis at the Tissue Scale

In this section, we identify the current approaches to modeling tumor vasculature at the tissue scale. We then describe quantitative imaging techniques for observing vascular changes over time at this scale and conclude by discussing how these quantitative measures can be integrated with mathematical models to make testable predictions.

### 4.1. Mathematical Modeling of Tumor Vasculature and Angiogenesis at the Tissue Scale

**Figure 4:**
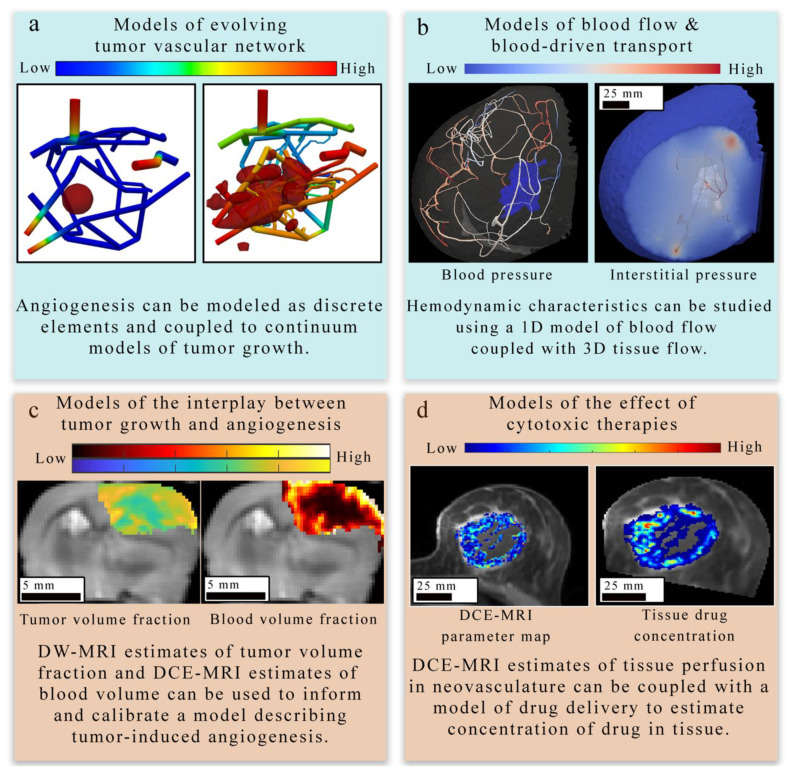
Overview of tissue-scale models of angiogenesis and vasculature. There are four main approaches to modeling tumor-induced angiogenesis and vasculature at the tissue scale level. (Panel **a**) provides an example of a discrete modeling approach [122,123] used to describe the evolving geometry of tumor vasculature in response to tumor growth. This simulation employs a 3D continuous multi-species tumor growth model coupled to a 1D discrete model of angiogenesis. The tissue domain initially features a small spherical tumor core, which grows in response to the changing vasculature network. The colors in the network show the nutrient volume fraction. (Panel **b**) displays how the function of existing tumor vasculature in the breast can be studied with computational fluid dynamics [33] to estimate hemodynamic properties of the vascular network. In (panel **c**), diffusion weighted (DW-) and DCE-MRI acquired in a murine brain tumor model (C6 glioma) are used to provide tumor volume fraction and blood volume fraction estimates to initialize and calibrate a model of tumor-induced angiogenesis. The model derived estimates of tumor and blood volume fraction are overlaid on an axial *T*_2_-weighted MRI through the center slice of the tumor. A coupled set of PDEs [32] are used to describe the proliferation, diffusion and death of tumor cells and the angiogenesis, diffusion, and regression of the vasculature. In (panel **d**), estimates of tissue perfusion in the breast derived from quantitative imaging are coupled with a mathematical model of drug delivery [86] and tumor growth to observe the effect of tumor vasculature on drug distribution and tumor response to treatment. Both the left and right images in (panel **d**) show quantitative maps of DCE-MRI parameters or drug concentration overlaid on an anatomical image acquired in the same plane. The right drug concentration map is an enlargement of the computational domain.

Similar to the cell scale approaches in Section 3.1, there are analogous continuous, discrete, and hybrid approaches that have been scaled up to describe angiogenesis and tumor-associated vasculature at the tissue scale. The choice of the modeling paradigm is highly influenced by the primary aim of the model and (potentially) the type of data used for validation. In this section, we identify four major areas of research at the tissue scale (shown in Figure 4) and discuss the modeling strategy or strategies applied to these areas. Broadly, these areas include: (1) representing the evolving geometry of the tumor’s vascular network (panel a in Figure 4) [29,82,87,88,119,120,121,122,123], (2) estimating the associated blood flow and vascular transport of substances (panel b in Figure 3) [81,88,122,123,124,125,126,127], (3) describing the mechanisms underlying the complex interplay between tumor growth and vasculature dynamics (panel c in Figure 4) [32,81,87,88,121,123,128,129], and (4) determining the effect of cytotoxic, targeted, and anti-angiogenic therapies on the tumor-associated vascular network as well as the tumor itself (panel d in Figure 4) [28,85,86,124].

#### 4.1.1. Models of Evolving Tumor Vascular Network

The first area Figure 4 of focus (panel a in Figure 4) bridges the cell to tissue scale by modeling the formation and evolution of tumor-induced angiogenic networks which are predominately modeled using a discrete (lattice-based and lattice-free), continuous, or hybrid strategy similar to those discussed in Section 3 [87,88,123,130,131,132]. Discrete approaches typically model individual TEC movement, while continuous approaches model the change in a spatially averaged, continuous variable (e.g., vasculature density or vascular volume fraction). Hybrid approaches combine the discrete and continuous approaches to provide a spatially resolved vasculature network, which can be mapped to a continuous domain to facilitate interaction with continuous elements of their mathematical modeling system (e.g., TAF or nutrients). One representative example by Frieboes et al. [87] applies a hybrid approach to describe angiogenesis coupled to tumor growth. Frieboes et al. use a lattice-free description of angiogenesis to describe TEC motion due to chemotaxis in response to TAF gradients and haptotaxis in response to fibronectin gradients. Once anastomosis occurs between two vessel branches, it was assumed that the now connected vasculature network could act as a source of oxygen and nutrients. Then, the distribution and availability of oxygen and nutrients directly influences tumor cell dynamics. Additionally, the discretized vasculature is spatially averaged to facilitate coupling to continuous elements within the model (i.e., TAF and fibronectin). The simulated tumor-induced angiogenic network produced a spatially heterogeneous distribution of oxygen and nutrients that resulted in phenotypic heterogeneity of tumor cells within the tumor.

#### 4.1.2. Models of Blood Flow and Blood-Driven Transport

**Figure 5:**
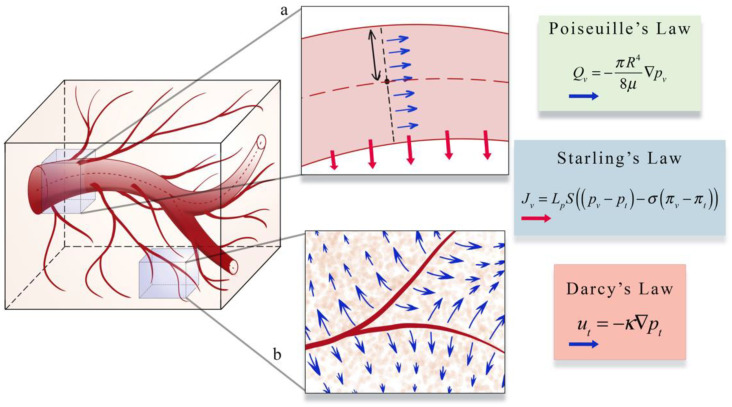
Illustration of a perfusion and transport model. Intravascular and interstitial flow is characterized by the laws of Poiseuille, Starling, and Darcy. Inset a illustrates Poiseuille’s and Starling’s law. Poiseuille’s law relates intravascular flow (*Q_v_*, blue arrows in inset **a**) to the radius of the vessel (*R*), the dynamic viscosity of blood *μ*, and the gradient of the intravascular pressure *p_v_*. Starling’s law relates the rate of extravasation (*J_v_,* red arrows in inset **b**) to the hydraulic conductivity of the vessel wall (*L_p_*), the vascular surface area (*S*), the reflection coefficient (*σ*), the vascular oncotic pressure (*π_v_*), and the interstitial oncotic pressure (*π_t_*). Inset b shows an illustration of Darcy’s law which relates the interstitial flow velocity (*m_t_*, blue arrows in inset **b**) to the interstitial tissue hydraulic conductivity *(κ*), and the gradient of interstitial pressure (*p_t_*). These three relations are found throughout the literature on the physical modeling of tumor associated vascular flow and angiogenesis.

The second area (panel b in Figure 4) focuses on estimating blood flow and transport within the vasculature and through the interstitial space. As described in Section 1, vascular flow has a profound influence on the dynamics of growth and therapeutic response of the tumor [8]. The modeling of vascular flow usually includes a description of flow in the blood vessels, along with its coupling with flow in the tissue through a mass flux at the capillary walls or at the terminal ends of larger vessels. Similar to the cell-scale models reviewed in Section 3, these phenomena can be modeled by discrete [33,87,88,122,123,126,130,131,132], continuous [128,129,133,134], or hybrid [127,135,136] approaches. In discrete vascular models both the pre-existing and the angiogenic vasculature are frequently approximated by a 1D network of connected straight cylinders with the flow in each cylinder simulated using the 1D Poiseuille law [33,87,88,122,123,126,130,131,132]. In continuous vascular flow models the vasculature is described with a spatially averaged, continuous variable (e.g., vasculature density or vascular volume fraction), and the transport of the substance of interest (e.g., drug or nutrient) through the interstitial space is described with a reaction-diffusion-advection model [128,129,133,134] describing the delivery, diffusion, and the transport of that substance due to bulk fluid flow. Hybrid vascular flow models [127,135,136] combine the discrete and continuum approaches; capillaries and smaller vessels are approximated with a continuum approach, whereas the large vessels are explicitly retained, and their flow is simulated as in discrete models. A formative example of blood flow and transport by D’Angelo et al. [131] describes an approach to couple a 1D discrete model of tissue vasculature with a 3D continuum model of interstitial transport. Blood flow through the vessel network follows Poiseuille’s law (which relates flow to vessel radius, pressure, and the viscosity of blood), and transport across the vascular walls is described by Starling’s law (which relates the extravasation rate to vessel permeability and the pressure difference between the vessel and the tissue). Interstitial flow is dictated by Darcy’s law, which relates flow to the pressure gradient and the hydraulic conductivity of the tissue. The approach by D’Angelo et al. allows the unique vasculature network structure to be preserved (and not reduced to a spatially averaged variable) while allowing for a coupling to a 3Dcontinuous model of the interstitial space. Figure 5 shows an illustration of these three foundational relations in modeling intravascular and interstitial flow.

#### 4.1.3. Models of Tumor and Vasculature Growth and Response to Therapy

The third and fourth areas focus on describing the mechanisms underlying the complex interplay between tumor growth and vasculature in the absence of treatment (panel c in Figure 4) [32,81,87,88,121,123,128,129] and during treatment (panel d in Figure 4). Many of the same discrete, continuum, and hybrid models of angiogenesis and vasculature network mentioned in the previous two areas are also applied to study the interplay between the tumor and vasculature with an increased emphasis on modeling the tumors themselves, as they trigger the angiogenic cascade, influence the development of the neovasculature, and are the ultimate beneficiaries of the angiogenic blood supply. At the tissue scale, models of tumor cell dynamics are typically captured in a continuous fashion by means of a PDE system [20,137,138,139]. This is most commonly achieved through either reaction-diffusion-advection equations or phase-field equations. Reaction-diffusion-advection equations describe the spatiotemporal dynamics of cell density (or tumor volume fraction) as a combination of random movement of cells via diffusion, directed movement of cells via advection, and reaction terms representing (for example) tumor cell proliferation, apoptosis, and cytotoxic effects due to treatments [32,86,127,128,129,134,138]. For example, Hahnfeldt et al. [139] developed a model of tumor volume dynamics as a function of the effective vascular support (or carrying capacity). The vascular influenced carrying capacity changes in response to stimulating effects (via tumor cells) and inhibitory effects (via endogenous and exogenous factors). This modeling formulation allowed the investigation of different anti-angiogenic therapies. Alternatively, phase field models may be used to describe the coexistence of a number of phases representing different tissue types (e.g., tumor and normal tissue) [140] and their interactions or transitions between each other. The spatial and temporal evolution is dictated by a free energy potential, which restricts mixing and penalizes spatial variation in individual phases, and several source terms describing the growth, death, response to treatment, and transition from one species to another (e.g., a proliferative tumor cell may transition to hypoxic tumor cell in response to scarce nutrients) [81,87,121,137]. The PDE governing the dynamics of each cell species is obtained by combining the mass flux, which is defined in terms of the gradient of the free energy potential [140], and the aforementioned source terms [81,87,121,137]. A detailed review of tumor growth modeling approaches can be found in [138,141].

An informative example of modeling the interplay between tumor and vasculature was proposed by Swanson et al. [129], who employed a continuum approach to model the transition of tumor cells between different phenotypes as a result of the vasculature density. Specifically, three tumor cell phenotypes were considered: prolific, hypoxic, and necrotic. Prolific cells were considered to be proliferative and mobile tumor cells in a normoxic or oxygen sufficient state. Alternatively, hypoxic cells were considered to be mobile tumor cells in a hypoxic or oxygen deprived state and could not proliferate. Cells initially begin as prolific cells and then transition to hypoxic cells once the relative fraction of vasculature (used as a surrogate for oxygen supply) is insufficient to support all the prolific cells. If the vasculature remains insufficient to support prolific and hypoxic cells, they eventually transition to necrotic cells. Vasculature growth is stimulated via the release of angiogenic factors from prolific and hypoxic cells. Using this coupled PDE system, the authors were able to recapitulate histological features of malignant progression (such as increased cellularity, hypoxia-induced angiogenesis, and necrosis) as observed in vivo. This multispecies model was also applied to simulate tumor response to anti-angiogenic therapy [133].

Vavourakis et al. [124] demonstrated an in silico method for modeling the influence of chemotherapies on tumor and vasculature dynamics using a model that characterizes tumor growth and therapeutic response, angiogenesis and vasculature remodeling, blood and interstitial flow, and the dynamics of key substances (e.g., TAF, oxygen, matrix degrading enzymes). The tumor-associated vasculature is modeled using a discrete approach, while the tumor is modeled using a continuum approach. The evolution of the concentration of cytotoxic drugs is modeled via continuous equations and accounts for several drug states (e.g., bound and unbound) as well as the different drug transport dynamics (e.g., advection and diffusion) in the bloodstream and the interstitial space [124,125,128]. Using their comprehensive framework, the authors were able to investigate the influence of drug properties (e.g., size and affinity), vessel porosity, the normalization of vessels, and treatment schedule on tumor regression. They observed that time-of-treatment was an important factor for low-affinity cytotoxic drugs and that high-affinity cytotoxic agents resulted in a large vascular normalization window that might enhance the delivery of subsequent chemotherapy doses.

As noted in Section 3.1., the choice of continuum, discrete, or hybrid modeling approaches is dependent on the desired goal or quantity of interest from the modeling exercise itself. Continuum models of angiogenesis, that use a spatially averaged variable to describe the tumor-induced vasculature (e.g., neovasculature density or volume fraction), provide a computationally tractable approach to explore the interplay between the tumor and supporting vasculature at both greater length and time scales compared to discrete models. At the tissue scale, this is an important consideration as modeling efforts are often investigating tumor growth and treatment response on the time scale of months to years. The main advantage of discrete models is that they can capture the precise changes in the angiogenic network and blood flow, thereby providing a better description of the transport of nutrients and drugs to the tumor region [33,87,88,122,123,124,125]. However, discrete models of angiogenesis may require the calibration of a large set of parameters as well as extensive computational resources to track both the existing and developing vasculature, and to couple the resolution of continuous and discrete phases in a multi-physics framework. Thus, discrete models are usually limited to small spatial scales and short time intervals (e.g., modeling the transition from avascular to vascular tumors). Hybrid models combine the advantages of both discrete and continuum approaches, as they retain the ability to represent large vessels via discrete methods resulting in a more accurate and patient-specific flow, while approximating the dynamics of tumor-induced capillaries through continuum approaches. Thus, hybrid models avoid explicitly tracking the evolution of every single branch in the angiogenic network independently, and therefore enable studying vascular tumor growth at various spatial and temporal scales. A final advantage for any of these approaches is the type of data available to calibrate or inform the model, discussed further in Section 2.2. Data that are able to resolve vessels may be more appropriate for discrete or hybrid modeling techniques, while imaging data that only return spatially averaged estimates of vascular volume are generally better suited for continuum modeling techniques.

### 4.2. Integrating Theory and Experimental Data at the Tissue Scale

Recent studies have proposed several promising approaches for integrating mathematical models with experimental imaging data at both the pre-clinical and clinical levels. In this section we identify approaches that focus on describing perfusion and delivery (Section 4.2.1) and treatment response (Section 4.2.2). The reader is referred to Table 3 for a summary of these approaches and the type of data used to inform the model.

#### 4.2.1. Applications to Estimate Perfusion and Delivery

Recent studies have provided important foundations on integrating imaging measurements of tumor-associated vasculature with mathematical models, which can provide a means to rigorously understand and predict tumor blood flow, interstitial transport, and angiogenesis. For example, d’Esposito et al. [80] performed fluorescence imaging to visualize tumor microvasculature in fixed tumor samples to inform a model of tumor perfusion. The segmented microvasculature was used to initialize the vasculature network for a computational fluid dynamic (CFD) model describing steady-state blood and interstitial flow. Using the CFD model, the authors estimated interstitial fluid pressure and velocity, blood flow and pressure, and the delivery of a widely used MRI contrast agent. Their CFD model predicted a heterogeneous spatial distribution of the contrast agent, which was validated against in vivo DCE-MRI. Similarly, Stamatelos et al. [47,48] applied a CFD model to a whole tumor microvasculature network imaged with ex vivo micro-CT imaging. Stamatelos et al. applied their model to study intravascular oxygenation, hemodynamics, and vascular morphology across eight breast tumor xenografts. Through this modeling framework, the authors demonstrated that the unique microvasculature network in an individual tumor contributes to both the inter- and intra-tumor heterogeneity.

Adhikarla et al. [83,84] developed a modeling workflow based on ordinary differential equations to simulate temporal changes in tumor vasculature and blood oxygenation. The microvasculature was initialized with micro-CT imaging, the tumor oxygenation status was calibrated with PET imaging data sensitive to hypoxia, and tumor growth was characterized by proliferation estimated from PET imaging data. These studies were able to use experimental data to provide physical conditions and domains for the mathematical modeling of tumor-related fluid dynamics. However, vasculature measurements from ex vivo imaging have limited clinical utility for diagnosis or prognosis because they require an invasive procedure that damages the system under investigation and, hence, cannot provide information on the remaining lesion or host tissue.

A non-invasive approach proposed by Wu et al. [33] applies a CFD model to the clinically available MR data. Wu et al. established a rigorous framework for integrating multiparametric MRI with a mechanism-based, biophysical model enabling the characterization of the hemodynamics associated with breast cancer on a patient-specific basis. Specifically, pre-treatment quantitative MRI data, including DCE-MRI and diffusion-weighted MRI, were employed to identify the patient-specific tissue geometry (e.g., tumorous, adipose, and fibroglandular tissues, along with vasculature) and properties (e.g., vascular permeability, interstitial hydraulic conductivity). These data were used to constrain a CFD modeling system, which coupled 1D blood flow with 3D tissue flow, enabling the characterization of hemodynamic characteristics, including blood flow rates, fluid extraction rate, interstitial pressure, and flow velocity. Using this approach, the authors observed significant differences in tumor-associated interstitial flow velocity, blood pressure, and vascular extraction rate between malignant and benign lesions.

#### 4.2.2. Applications to Treatment Response

The treatment efficacy of systemic therapies administered intravenously relies on the delivery of drugs through the bloodstream, which is highly dependent on the vascular structure and associated perfusion. Additionally, multiple pre-clinical and clinical studies have shown that anti-VEGF therapy changes tumor vasculature towards a more “mature” or “normal” phenotype, thereby improving the delivery and efficacy of concomitant chemotherapies [142]. Therefore, the use of data-driven modeling to evaluate angiogenesis is a promising means to assess and predict tumor response to therapies. The approach proposed by Titz et al. [143] employed a continuum model to simulate tumor and vasculature responses to anti-angiogenic therapy. Pre-treatment PET measurements of cellular proliferation and hypoxia were used to initialize the simulation and estimate model parameters. In their simulations, hypoxic tumors released TAF or VEGF to stimulate endothelial cell proliferation and an increase in microvessel density. The estimated microvessel density was used to estimate the average voxel oxygenation. The model parameters describing cellular and vascular proliferation were adjusted to minimize the error between the measured oxygenation from PET and the model-estimated oxygenation. Using this modeling framework, the authors estimated the response to anti-angiogenic therapy and demonstrated that anti-angiogenic therapy could be personalized based on the initial levels of VEGF within the tumor.

The influence of vasculature on tumor response to radiotherapy was considered by Hormuth et al. [32,85,144], who used a coupled PDE-based model of tumor growth and angiogenesis in a murine model of glioma. Quantitative MRI collected before and after radiation therapy were used to initialize estimates of tumor cellularity (from diffusion-weighted MRI [145]) and blood volume fraction (from DCE-MRI) as well as to calibrate model parameters. The two PDEs were coupled by assuming the blood volume fraction was linearly related to the maximum amount of tumor cells that could be supported in a given voxel as determined in a previous study in the absence of treatment [32]. Similarly, a previous study assessing the validity of 39 models of tumor growth and radiotherapy response [137] was used to guide modeling of tumor and vasculature response to radiotherapy. When response to radiotherapy was considered, Hormuth et al. observed that spatially varying the efficacy of radiotherapy as a function of local blood volume fraction also improved predictions of tumor response [85,144].

A similar approach by Jarrett et al. [86] modeled the action of neoadjuvant therapy on breast cancer in a patient-specific setting. Jarrett et al. extended the PDE-based model of breast cancer response to neoadjuvant therapy proposed by [146] by including the effects of drug delivery. The tumor response model was initialized with patient-specific diffusion-weighted MRI data and drug delivery estimated using DCE-MRI data. The literature estimates of the drug concentration in the plasma were coupled to patient-specific estimates of vessel permeability and perfusion to simulate the intra-tumor distribution of neoadjuvant therapies. This study demonstrated the plausibility of using DCE-MRI data as a means to estimate drug delivery on a patient-specific basis in predictive models and represents a pivotal step towards the goal of achieving individualized prediction of tumor response to therapy. Additionally, this work has been extended by calibrating the model with follow-up images collected during neoadjuvant therapy [28]. The extended model enables a rigorous prediction of patient-specific response to the prescribed treatment, thereby providing novel opportunities to identify alternative treatment regimens for patients with inadequate response to standard-of-care treatments.

## 5. Opportunities for Multiscale Modeling of Angiogenesis

The formation of blood vessels during tumor growth is a process that spans multiple spatial and temporal scales. For instance, signaling pathways activated in endothelial cells in response to the binding of TAFs to its receptor occur at the subcellular scale, the movement of TECs as well as cell–cell and cell–extracellular matrix interactions happen at the cellular scale, and blood flow along with the delivery of nutrients and therapeutics occurs at the tissue scale [147,148,149]. Therefore, each scale provides a complementary picture of the formation of the tumor vasculature. Additionally, while signaling pathways and TEC motion may feature fast mechanisms on the order of milliseconds to seconds, the formation of fully functioning new vessels may take days and the vascular-induced changes in tumor growth may occur over weeks [150]. Thus, to fully characterize the complexity of angiogenesis, multiscale mathematical models that combine the description of biological processes underlying the formation of tumor-induced neovasculature at multiple scales are needed [24,76,141,147,148,149,150,151,152,153,154,155]. Some models of angiogenesis already include a multiscale component. For example, Vilanova et al. [29,101] modeled TEC motion along with capillary formation, which occur at the cell and tissue scale, respectively. Furthermore, Vavourakis et al. have proposed a multiscale model including interstitial and vascular transport, ECM degradation, explicit vessel formation and remodeling, tumor-induced tissue deformation, and the dynamics of drug distribution, binding, and internalization [88,124]. Ultimately, these models constitute a promising approach to precisely predict tumor vascularization, vascular-induced changes in tumor dynamics, and therapeutic outcome. For example, by modeling the delivery of drugs in the vasculature and interstitial space, their interaction with tumor cells at the cellular scale, and the specific action of the drugs on signaling pathways at the subcellular scale, multiscale models could enable the exploration of the cascade of effects of different treatment strategies [125,156].

It is important to acknowledge that while the use of high-performance computing techniques is becoming more common, solving multiscale models of angiogenesis is still computationally intensive and one of the fundamental challenges in model development. Multiscale models are generally hybrid models that combine systems of ordinary differential equations (e.g., signaling pathways) and PDEs (e.g., blood flow, drug delivery, and tissue heterogeneity) with discrete models (e.g., cell-cell iteration, TEC movement). The coupling of these models, while considering the stochastic nature and different time scales of many angiogenesis processes, contributes to the challenge in developing computationally tractable numerical solvers to perform computer simulations. There is also an abundance of plausible models that can be applied to represent mechanisms at each scale. Thus, selecting the most appropriate model is a great challenge and techniques are needed to systematically evaluate the validity of models [137,157]. While scale-specific model selection has already been investigated [137,158], the selection and combination of models at different scales is yet to be explored. Finally, due to the model complexity and large number of parameters in multiscale formulations, there is a fundamental challenge to obtain sufficient data to calibrate and validate these models [137]. While one can still draw useful conclusions from qualitative experiments [156], the model parameters must be initialized and constrained with patient-specific data to make clinically relevant predictions [24]. However, even with the advances in medical imaging, with the current clinically available data it is impossible to assign values to every parameter in multiscale models.

## 6. Future Directions

The recent convergence of time-resolved imaging and mathematical modeling is beginning to enable in silico investigations into the spatial-temporal evolution of vasculature structure and function that can then be tested in the in vitro and in vivo settings. There are several promising avenues for future research to further develop image-driven biologically based models of angiogenesis. First, there is an abundance of imaging techniques at the tissue scale that can quantify tumor vasculature (Section 2.2). Several of these techniques are routinely collected in the standard-of-care setting, but the quantitative analysis of these data is less common outside of the research setting. To enable the widespread use of tissue-scale models of angiogenesis, these imaging analysis techniques need be translated into the clinic [159,160]. Additionally, acquisition and analysis protocols to reduce uncertainty in the imaging measurements need to be developed. We [161,162,163] and others [164,165,166,167] have begun to demonstrate that quantitative imaging techniques to quantify tissue vascularity can be performed with high accuracy and precision. Furthermore, we have shown that certain MRI measures can be collected with high quality in the community setting (and not in a research or academic setting) using widely available hardware [28].

Second, the modeling of angiogenesis at the cell scale has been predominantly validated by experiments in a retrospective manner, rather than first informing or calibrating the model with longitudinal, time-resolved, data and then performing a prospective validation. However, there are limitations in both the experimental and computational techniques needed to effectively calibrate these models. For the microscopy-based approaches, phototoxicity or limitations in the number of fluorescent markers (or assays) may limit the duration of experiments and reduce the number of observed species, respectively. In addition, stochasticity in both observed endothelial cell movement and model implementations (e.g., discrete or hybrid models of angiogenesis) of endothelial cell movement results in an additional challenge in parameter estimation.

Finally, as the structure and function of vasculature fundamentally influences the efficacy of systemic and radiation therapies [8,168], and therefore patient outcomes, a practical understanding of a patient’s vasculature dynamics could be leveraged to identify improved therapeutic regimens. More specifically, we posit that image-driven modeling frameworks could be used to investigate systemic drug delivery, radiotherapy efficacy, and the identification of optimal therapeutic regimens [17,169]. The current standard-of-care treatment regimens are the result of large, expensive, and time-consuming clinical trials designed to assess treatment efficacy in a population of patients rather than identifying the optimal regimen for an individual patient. An in silico trial system may enable systematic evaluations of therapeutic regimens for individual patients based on a “digital twin” [17,170,171] of a patient’s unique tumor and vasculature network. Several promising modeling approaches have investigated optimizing chemotherapy based on imaging [28,172,173] or genomic data [174,175]. Preliminary efforts by Jarrett et al. [28] and Wu et al. [173], which include information about drug delivery, vasculature function, and tumor cell distribution in their modeling framework, were able to identify protocols that outperform a standardized dosing regimen. These modeling techniques could be integrated with optimal control theory [169] to provide a systematic approach to personalizing therapeutic regimens that improve therapeutic efficacy as well as reducing side-effect toxicity. This is particularly important for novel therapeutics and immunotherapy where there are substantial efforts at developing the mathematical theory [176,177,178] to characterize treatment response, but limited longitudinal imaging studies of the effects on the tumor and associated vasculature. One challenge for applying this image-driven framework is the parameterization of the effect of these novel therapeutics on a patient’s tumor or vasculature to determine the optimal regimen. Thus, without the pre-requisite data we are only able to hypothesize treatment effects. By combining experimental time-resolved imaging data with practical, validated, models of tumor growth and angiogenesis, there is a promising opportunity for precise, clinically relevant forecasts of patient-specific therapeutic response, which, in turn, may fundamentally shift (and improve) how patient care is delivered.

## 7. Conclusions

In summary, the integration of biologically-based mathematical modeling of tumor vasculature and angiogenesis with time-resolved experimental data promises to enable further understandings of angiogenesis from the cell to tissue scales. Models validated by experimental data, could then be used to generate testable hypotheses or predict the spatial-temporal evolution of the tumor and its associated vasculature. Furthermore, at the clinical level mathematical models initialized and constrained by quantitative imaging techniques could produce timely and actionable forecasts of tumor growth and response that could help guide clinical decisions and fundamentally improve patient care.

## Figures and Tables

**Table 2:** Examples of studies integrating imaging data with mathematical modeling at the cell scale.

Paper	Modeling Approach	Scale	Use of Data
Perfahl 2011 [76]	Discrete	Cell-tissue	Microscopy used to initialize vasculature network
Xu 2020 [81]	Hybrid	Cell-tissue	Photoacoustic imaging was used to initialize vasculature network
Stepanova 2021 [77]	Hybrid	Cell	Agent-based model was calibrated against in vitro assays
Phillips 2019,2020 [18,79,100]	Discrete	Cell	Time-resolved microscopy was used to initialize and calibrate an agent-based model

**Table 3:** Examples of studies integrating imaging data with mathematical modeling at the tissue scale.

Paper	Modeling Approach	Scale	Use of Data
d’Esposito 2018 [80]	Continuum	Tissue	Whole tumor imaging was used to initialize vasculature network, perfusion model validated against DCE-MRI
Stamatelos 2019 [48]	Continuum	Tissue	Whole tumor microscopy was used to initialize tumor vasculature
Adhikarla 2012, 2016 [83,84]	Discrete	Tissue	CT data was used to initialize vasculature network, model parameters were calibrated against PET measures of hypoxia
Wu 2020 [33]	Continuum	Tissue	DCE-MRI used to initialize breast vasculature
Titz 2012 [143]	Continuum	Cell-Tissue	PET estimates of oxygenation and proliferation were used to initialize tumor simulation and calibrate model parameters
Hormuth 2019,2020 [32,85]	Continuum	Tissue	Time-resolved DCE-MRI to calibrate and validate models
Jarrett 2018, 2020 [28,86]	Continuum	Tissue	Time-resolved DCE-MRI used to estimate drug delivery

## Data Availability

Not applicable.

## Abbreviations

MRI, magnetic resonance imaging; CT, computed tomography; PET, positron emission tomography; MRA, magnetic resonance angiography; DCE-dynamic contrast-enhanced; PDE, partial differential equations; TAF, tumor angiogenic factors; TEC, tip endothelial cell; ECM, extra-cellular matrix; CPM, cellular Potts models; VEGF, vascular endothelial growth factor; DW, diffusion weighted; CFD, computational fluid dynamic; ODE, ordinary differential equation.
